# Retrograde Transvenous Thoracic Duct Embolization for Tubercular Chylothorax

**DOI:** 10.7759/cureus.62010

**Published:** 2024-06-09

**Authors:** Prachita Agrawal, Pankaj Banode, Shubham Agrawal, Vadlamudi Nagendra

**Affiliations:** 1 Department of Interventional Radiology, Jawaharlal Nehru Medical College, Datta Meghe Institute of Higher Education and Research, Wardha, IND; 2 Department of Neurology, Sawai Man Singh Medical College, Jaipur, IND

**Keywords:** mycobacterium tuberculosis, computed tomography, intercostal drainage, thoracic duct embolisation, chylothorax

## Abstract

This case study documents the clinical profile of a 27-year-old male patient who visited the medical facility two months ago with complaints of dry cough, fatigue, weight loss, and occasional fever. He had been treated for ascites and pleural effusion in the hospital before presentation and returned with an intercostal drain in place. A detailed examination revealed symptoms of respiratory disorders, including fluid in both lungs, fever, and dyspnea. His fluid levels showed multiple deviations from the normal range, according to the report's findings and lab test results. It was determined that the patient had chylothorax, which resulted from hemophagocytic lymphohistiocytosis (HLH) and abdominal tubercular lymphadenopathy. His anti-tubercular treatment (AKT4) was initiated, along with octreotide for his management. Initial management included non-invasive ventilator (NIV) support, intravenous antibiotics, nebulization, and an intercostal chest drain (ICD). Later, the patient underwent retrograde transvenous thoracic duct embolization (TDE) using N-butyl cyanoacrylate (NBCA) glue. The removal of the drainage tube and the patient’s stable discharge were made possible through regular monitoring and collaboration between specialists.

## Introduction

A chyle deposit in the pleural cavity is known as chylothorax. Healthcare workers face a significant challenge when dealing with chylothorax. Chyle, a milky liquid secreted by the intestinal lacteal system, consists of small and medium-chain triglycerides in the meal that are swiftly changed by intestinal enzymes into free fatty acids, which are then taken up by the portal circulation. However, the large molecules present in complex long-chain triglycerides are insoluble in intestinal lipases. In the jejunum, they combine with cholesterol, cholesterol esters, and phospholipids to form chylomicrons. The lymphatic system of the small intestine absorbs these large molecules, which combine to form the chyle [1]. The lymphatic system transfers around 2.4 liters of chyle every day. Rupture or damage to the thoracic duct can cause a rapid accumulation of fluid in the pleural area [2]. To achieve the best possible patient outcome, our case management entailed treating the primary condition, providing proper cautious nutritional support, and taking suitable measures such as thoughtful diaphragmatic drainage [3]. Chylothorax can be triggered by trauma, surgical and nonsurgical, lymphomatous and non-lymphomatous cancer, and a variety of benign disorders including infections like sarcoidosis, tuberculosis, filariasis, and others [4].

Thoracic duct embolization (TDE) for chylothorax was safe and effective: a retrospective analysis of 26 patients showed that low output chylothorax (<1 L/day) was conservatively managed by diet modification, but high output (>1 L/day) was managed surgically with thoracic duct ligation [5]. Non-traumatic chylous leaks were relatively rare and could arise from any condition that led to lymphatic blockade, such as lymph vascular disease (lymphangiomatosis, Gorham disease), systemic disease (sarcoidosis, Behcet disease), malignancy (lymphoma), or congenital factors or idiopathic causes [6].

## Case presentation

Patient information

A 27-year-old male presented to our medical institution with complaints of a dry cough, pervasive fatigue, significant weight loss, and sporadic bouts of fever that had persisted for two months. Following a 15-day therapy at another institution for pleural effusion, he was referred to our hospital after the placement of an intercostal drainage (ICD) tube to reduce the effusion.

Physical examination

General examinations revealed a fever of 38.5°C, tachycardia at 120 beats per minute, and blood pressure of 110/80 millimeters of mercury (mmHg), considered normotensive. Chest auscultation revealed bilaterally reduced breath sounds.

Medical/surgical history

**Figure 1:**
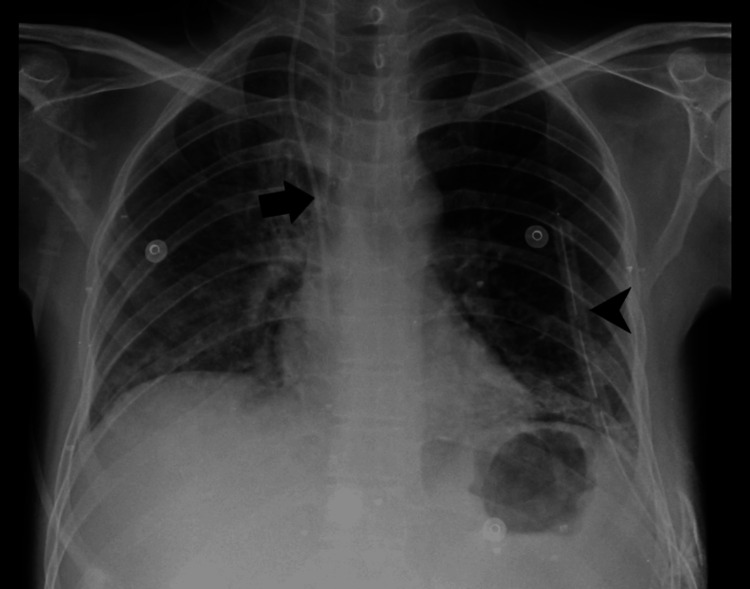
Heterogeneous opacity in bilateral mid and lower lung fields. An arrow denotes the right central line. An arrowhead shows the left intercostal drainage tube.

A previous chest X-ray revealed heterogeneous opacities in both lung fields, as well as bilateral pleural effusion, with a left-sided ICD inserted. Following admission to the ICU and undergoing non-invasive ventilation (NIV), the patient's chest X-rays revealed respiratory distress and bilateral lung region haziness, as illustrated in Figure 1.

Investigation

**Figure 2:**
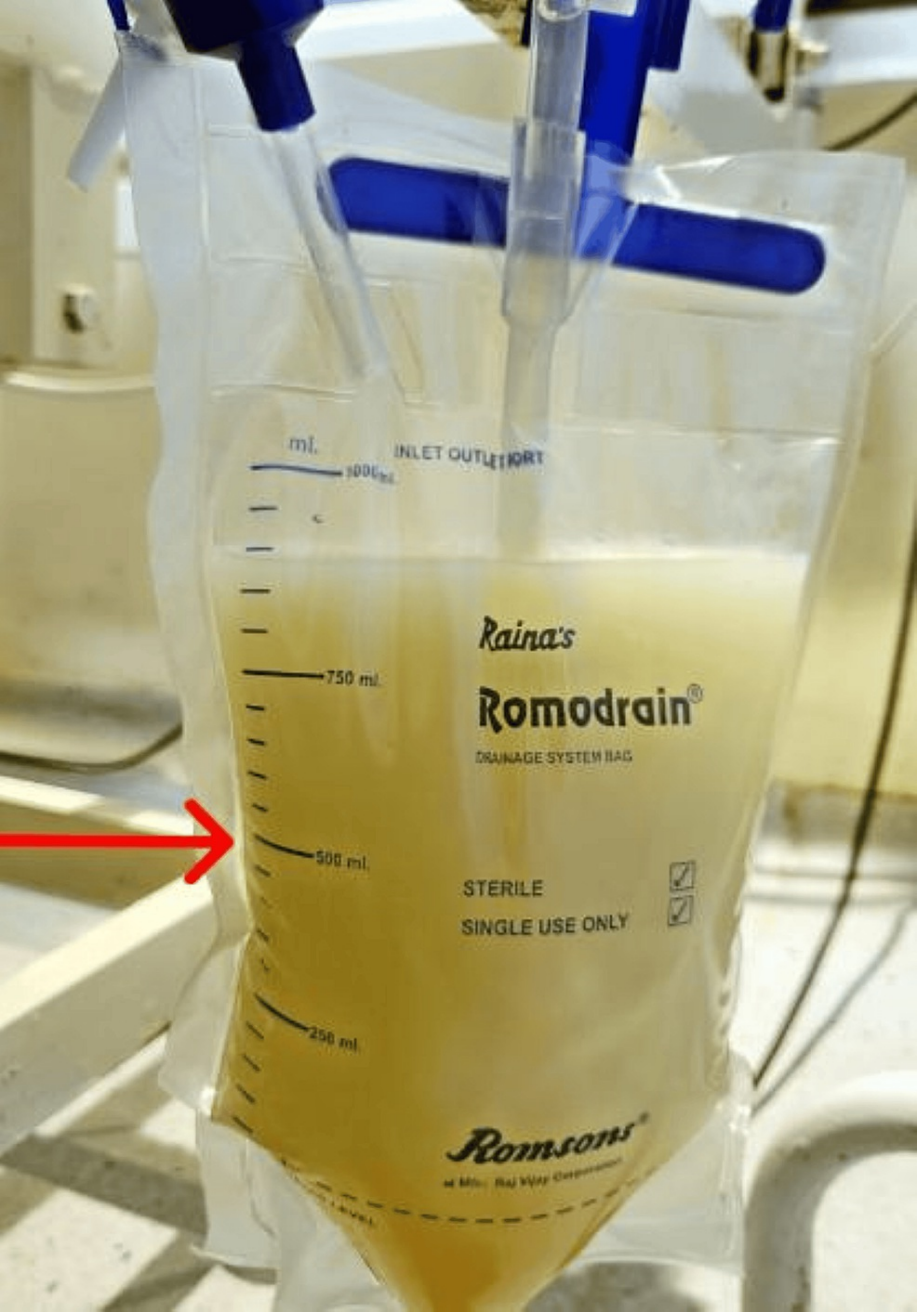
The milky white appearance of pleural fluid.

The patient was recommended for diagnostic thoracentesis and paracentesis. The report revealed a milky white appearance of the pleural fluid, as seen in Figure 2.

**Figure 3:**
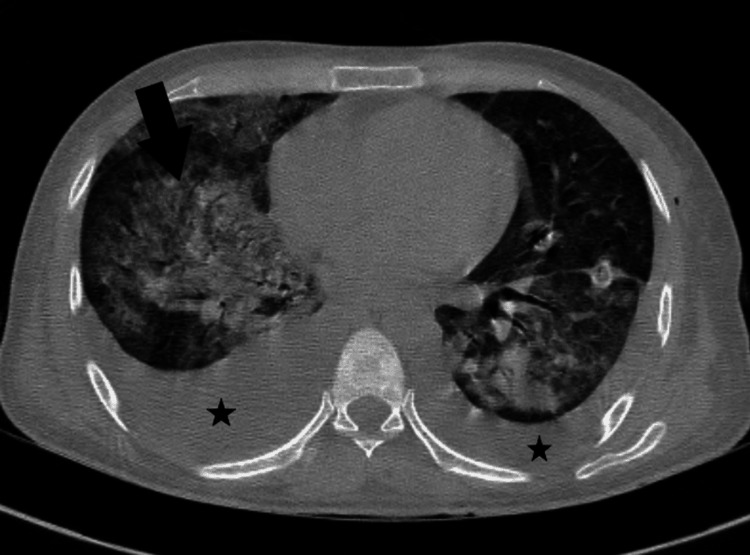
Non-contrast CT thorax axial image showing bilateral pleural effusion (star), right more than left, with collapse consolidation of underlying lung parenchyma. An arrowhead indicates diffuse ground glass opacities.

Moderate pleural effusion was present on the left side and minor effusion on the right, along with collapse consolidation of the underlying pulmonary parenchyma and bilateral basal atelectasis, as shown in Figure 3.

Computed tomography of the thorax was advised. It revealed extensive ground-glass opacities in all segments of the bilateral lung field, along with thickening of interlobular septae and signs of acute respiratory distress syndrome. Computed tomography of the abdomen showed multiple enlarged necrotic and reactive conglomerate retroperitoneal lymph nodes.

The patient underwent an ultrasound-guided biopsy of the paraaortic lymph node, which identified Mycobacterium tuberculosis infection (MTB) via GeneXpert and revealed necrotizing granuloma on cytology.

Treatment

The patient was given anti-Koch's medication, which includes isoniazid, rifampicin, pyrazinamide, and ethambutol, due to high pleural fluid adenosine deaminase (ADA) levels and findings from the paraaortic lymph node biopsy. The gastroenterologist prescribed octreotide for three months, at a dose of 50 micrograms three times daily. After conservative treatment failed, the patient underwent percutaneous TDE.

**Figure 4:**
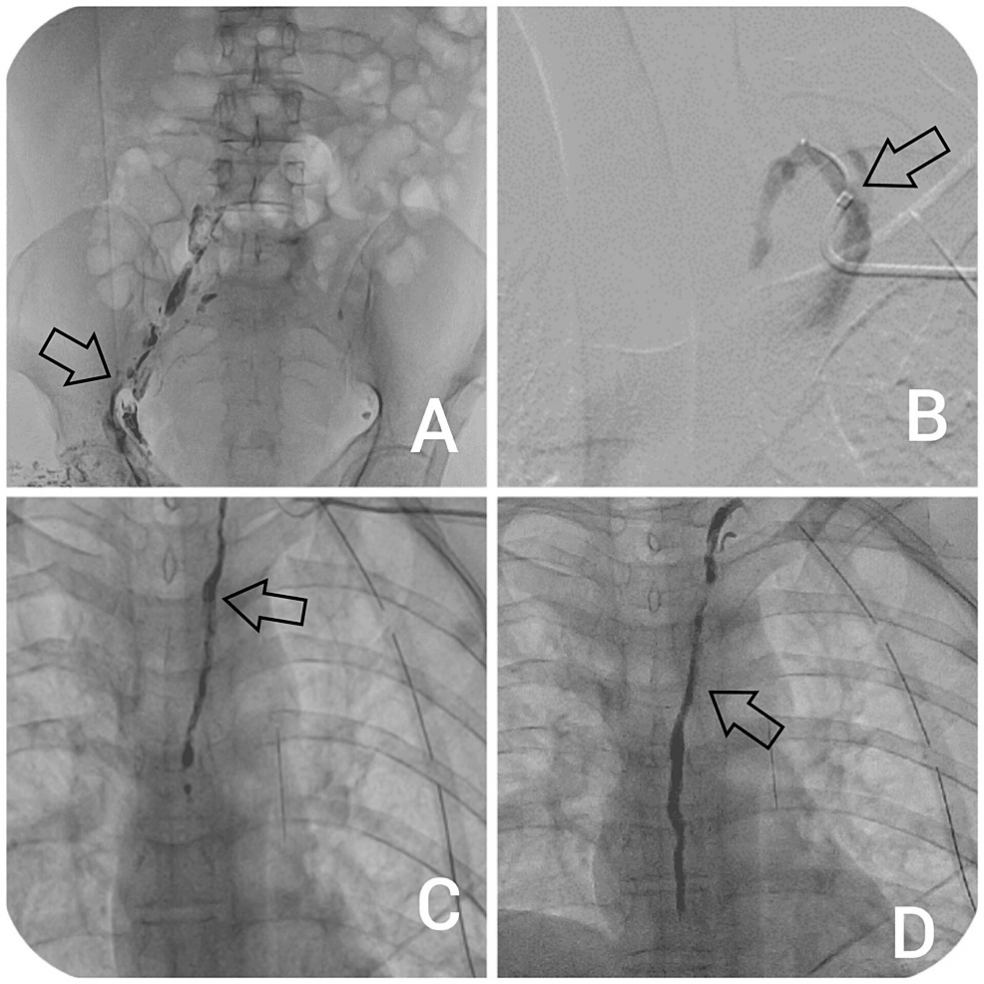
DSA images during the embolization procedure. A - Arrow indicating right iliac intranodal lymphangiography. B - Arrow showing the thoracic duct drainage site at the jugular and subclavian vein junction. C - Contrast (arrow) in the main thoracic duct. D - Embolization of the main thoracic duct with N-butyl cyanoacrylate (NBCA) glue (arrow). DSA: Digital subtraction angiography.

Intranodal lymphangiography was performed as shown in Figure 4A. Under ultrasound guidance, a 25G spinal needle was used to puncture the hilum of an inguinal lymph node, and an oil-based contrast agent (Lipiodol) was administered at a rate of 1 to 2 mL every 5 minutes, up to a total of 6 mL. The leak went undetected as Lipiodol did not enter the main thoracic duct, potentially being washed out into venous channels.

The patient was advised to undergo transvenous retrograde TDE. A venogram was obtained after gaining ultrasound-guided access to the left basilic vein and inserting a 5F sheath. A 5F catheter was placed into the brachiocephalic vein to investigate the thoracic duct drainage site near the jugular vein-subclavian vein junction. The contrast was injected, and the flow pattern was evaluated using fluoroscopy. A single long vessel with an extremely sluggish flow was found and selectively catheterized as shown in Figure 4B.

A Progreat 0.27 (Terumo) microcatheter with a 0.014 microwire was placed a few centimeters into the main thoracic duct. The contrast was injected to confirm proper entry into the thoracic duct and to demonstrate its flow pattern, as shown in Figure 4C. The microcatheter was advanced into the lymphatic system with a 0.014-inch guidewire. Further examination demonstrated active contrast extravasation suggesting the site of the leak; the microguidewire and microcatheter were inserted distal to the leakage site and the thoracic duct was embolized using a 75% N-butyl cyanoacrylate (NBCA) glue and Lipiodol mixture, as shown in Figure 4D.

Follow-up

**Figure 5:**
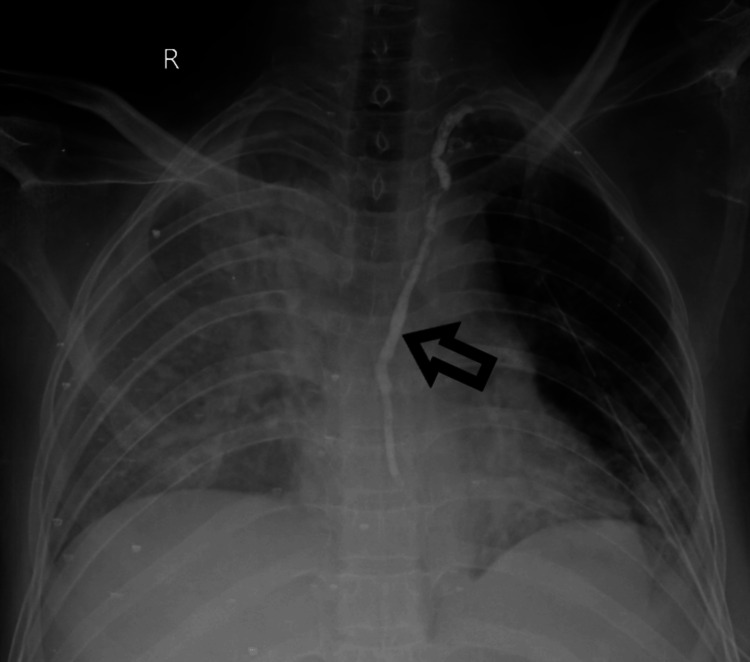
Post-embolization chest X-ray PA view showing hyperdense material in the main thoracic duct (arrow), suggesting complete embolization with NBCA glue and the absence of pleural effusion. PA: Posteroanterior; NBCA: N-Butyl Cyanoacrylate.

**Figure 6:**
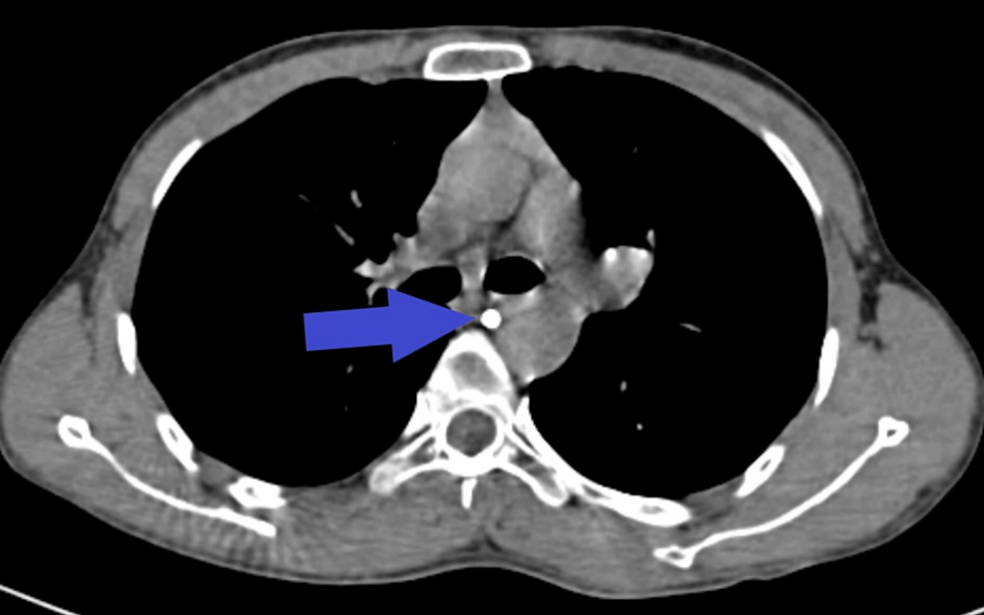
Post-embolization plain CT chest showing hyperdense material (arrow) in the main thoracic duct, suggestive of NBCA glue. NBCA: N-Butyl Cyanoacrylate.

Chyle output rapidly dropped during the TDE and eventually stopped on day 20 of recovery. Consequently, anti-tuberculosis treatment (ATT), octreotide, and dietary changes were recommended. The intercostal drainage (ICD) was removed after decreased drainage and improvements in consecutive chest X-rays, allowing the patient to be discharged in stable condition. The patient's post-interventional course was uncomplicated, and numerous subsequent chest X-rays revealed no effusion, as shown in Figures 5-6.

## Discussion

Chylothorax, an acute condition linked to immunosuppression and malnutrition, can lead to significant mortality. Bartolet initially described chylothorax in 1633, and Quinke reported the first case in 1875. The most common cause of chylothorax is trauma; however, tumors and other factors may also contribute. The thoracic duct's length renders it susceptible to damage. Chylothorax can result from the subclavian vein becoming mobilized during esophageal, pulmonary, or cardiovascular surgery. It can also be observed after trauma such as road traffic accidents, falls from height, and compression injuries to the abdomen and thoracic cavity. Malignancy is the second most common cause reported; 75% of cases are lymphomas, while leukemias and bronchogenic carcinomas are also seen, though rarely [7].

TB lymphadenopathy is an uncommon cause of chylothorax, affecting both immunocompetent and immunocompromised individuals. There are very few case reports in the literature that mention chylothorax as a consequence of tuberculous lymphadenitis. When there is mediastinal lymphadenopathy due to tuberculosis, lymph nodes can invade or impair the cisterna chyli and/or thoracic duct. This can raise pressure within the surrounding lymphatic system and cause chylous material to seep into the pleural cavity. Chylothorax, an uncommon clinical disease, is defined by low cholesterol and high triglyceride (TG) values in a milky pleural aspirate [8]. The majority of TB-chylothorax cases respond favorably to anti-Koch therapy and dietary changes, with only 17.1% requiring thoracic duct surgery [9].

In the present case, the antegrade technique failed, and retrograde transvenous TDE was performed successfully to treat the tubercular chylothorax. Compared to the percutaneous transthoracic or abdominal method or surgical thoracic duct closure, the retrograde transvenous approach is a less invasive and more successful treatment option. Inflow of embolic material into the pulmonary artery is the most frequent complication of this retrograde procedure. The issue that occurs when lymph nodes have undergone substantial surgical resection is that lymph fluid flow becomes impeded, making it impossible to perform a transabdominal puncture, and lymphangiography cannot locate the site of the thoracic duct injury. The biliary system, intestines, and arteries are among the organs that could be injured during a transabdominal procedure [10].

The antegrade TDE involves using ultrasound guidance to puncture the inguinal lymph node and has a technical success rate of about 70% [11]. Because the thoracic duct's ostial valve is typically challenging to image and the subclavian vein and thoracic duct confluence is difficult to cannulate because of the variety of venous anomalies, the retrograde transvenous procedure may be technically more difficult. Moreover, the thoracic duct's valves and variations may make it impossible for the contrast agent to reach the damaged site. The drainage site of the thoracic duct should be located using lymphangiography from the inguinal lymph nodes when a retrograde venous route is performed. In the event that inguinal lymph node lymphangiography is not possible, magnetic resonance imaging lymphangiography should be performed in advance to locate the vein and thoracic duct [10].

For traumatic chylothorax, percutaneous TDE has proven to be a less invasive and highly effective option compared to conservative or surgical approaches. However, in our case, even though non-traumatic TDE was performed, it yielded good results. Patients with chylothorax were often treated conservatively with total parenteral nutrition, a low-fat diet, octreotide, and pleural drainage. Previously, surgical thoracic duct ligation was recommended for patients with high-output chylothorax (>1000 mL/day) who had failed conservative treatment [12].

To diagnose chylothorax and perform TDE, a catheter was inserted into the thoracic duct, and various procedures were documented. Cope et al. identified transabdominal antegrade access as a prevalent method. The thoracic duct was catheterized by a microcatheter, which navigated multiple organs after a needle puncture established this treatment [13]. In 1-4% of esophageal surgeries, there was a high-output chylothorax due to a thoracic duct leak. The initial symptom was pleural effusion or persistent drainage from a chest tube. The diagnosis was confirmed by a triglyceride level in the pleural fluid greater than 110 mg/dL. Previously, full parenteral nutrition or a medium-chain fatty acid diet were used as conservative treatments for low-output chylothorax (<1000 mL/day). High-output chylothorax typically requires open or video-assisted early surgical ligation [12].

## Conclusions

In conclusion, this case report discussed the successful treatment of non-traumatic chylothorax in a tuberculosis patient using retrograde transvenous TDE. This treatment stood out as a less invasive and more effective alternative to conventional surgical procedures. Conventional thoracic duct ligation, commonly employed when antegrade procedures failed to discover the leakage point, carried a higher risk and required a longer recovery period. The retrograde transvenous technique was a promising option with fewer complications and a faster recovery time, indicating its potential as a first-line treatment. Therefore, clinicians should consider retrograde transvenous TDE before resorting to more invasive surgical approaches for addressing non-traumatic chylothorax, especially in cases where the leakage point remains undetected with antegrade procedures. This development has the potential to greatly enhance patient outcomes and lessen the burdens of more invasive procedures.
